# Evaluation of a hospital-based day-structuring exercise programme on exacerbated behavioural and psychological symptoms in dementia - the exercise carrousel: study protocol for a randomised controlled trial

**DOI:** 10.1186/s13063-015-0758-2

**Published:** 2015-05-26

**Authors:** Tim Fleiner, Wiebren Zijlstra, Hannah Dauth, Peter Haussermann

**Affiliations:** Institute of Movement and Sport Gerontology, German Sport University, Cologne, 50993 Germany; Department of Old Age Psychiatry, LVR-Klinik Köln, Köln, 51109 Germany

**Keywords:** Dementia, RCT, Old age psychiatry, Exercise, BPSD, Circadian rhythms, Rest-activity-cycles, Body-fixed motion sensors

## Abstract

**Background:**

Conceptual reviews and observational studies describe a link between physical inactivity and behavioural disturbances in people with dementia. Consequently, treatment of these symptoms requires physical activation and pharmacological or physical immobilization should be avoided. The few trials that have been conducted in inpatient dementia care to investigate the effects of exercise on behavioural and psychological symptoms revealed inconsistent results. Due to a lack of evidence, there is a paucity of recommendations for physical activation in this stage of care. Therefore, this trial seeks to investigate the effects of a day-structuring exercise programme on behavioural and psychological symptoms as well as on circadian rhythms of patients with dementia, hospitalized because of their behavioural and psychological disturbances.

**Methods/Design:**

A single-centre randomised controlled trial will be conducted in three special dementia care units of an old age psychiatry hospital. Enrolled patients will receive either a 2-week exercise programme, or a 2-week social stimulation programme in addition to usual care. Due to the provision of four day-structuring exercise-sessions in the course of an intervention day, the exercise programme for the study group is called *exercise-carrousel*. Baseline and post-intervention assessment for the primary outcome variable - the overall effects on behavioural and psychological symptoms - will be measured by the Alzheimer's disease Cooperative Study-Clinical Global Impression of Change. The following objectives are set up as secondary outcomes: dimensions of the behavioural and psychological symptoms of dementia (BPSD) and caregiver burden, routine and on-demand psychotropic medication, patients’ motor behaviour, diurnal cortisol-levels from saliva probes and brain-derived neurotrophic factor-levels from blood serum.

**Discussion:**

In order to be regarded as an important treatment option for behavioural and psychological symptoms, physical activation in inpatient hospital dementia care requires more evidence and appropriate recommendations. Respecting hospital routines and the intra-daily variability of the patients’ motivation and behavioural disturbances in the provision of exercise sessions could lead to higher exercise adherence and better effects on patients’ behavioural and psychological symptoms than former trials have presented. The concealment of allocation throughout the trial and the rating of individual exercise exertion present the key challenges and main limitations of this trial.

**Trial registration:**

DRKS00006740 (German Clinical Trial Register, date of registration: 28 October 2014).

## Background

The treatment of behavioural and psychological symptoms of dementia (BPSD) is a key challenge in inpatient dementia care [1, 2]. During the course of the disease nearly every patient suffers from an exacerbation of behavioural and psychological symptoms. Due to extreme caregiver and environmental burden, such phases often lead to admission into specialised Dementia Care Units (DCU) in old age psychiatric departments.

The application of antipsychotic medication is a standard treatment method for behavioural disturbances in inpatient dementia care. Owing to potential side effects of neuroleptics [3], clinicians seek non-pharmacological approaches for the treatment of BPSD [4]. A conceptual review [5] and an observational, cross-sectional trial [6] describe a direct link between physical inactivity and increased behavioural disturbances. Considering these findings, more physical activation in DCU is recommended, possibly leading to a reduced use of sedative medication and physical constraints. This non-pharmacological approach shows the positive effects of physical activation and may help to reduce critical side-effects of psychopharmacotherapy.

Unlike the good evidence for exercise as a key-factor in the prevention of dementia [7–9], research focussing on the effects of exercise in dementia care is in its ‘infancy’ [10]. While exercise trials focussing on functional performance and activities of daily living show promising results [11, 12], there is a paucity of clinical trials investigating the effects of exercise on BPSD. While trials in long-term nursing home care reveal promising effects on some BPSD - especially on mood, agitation and circadian disturbances - there are only a few clinical studies in inpatient hospital dementia care with consistent results [13]. The Cochrane Review by Forbes *et al.* [11] includes only one trial investigating the effects of exercise on BPSD [14]. This 1-year nursing-home exercise trial, conducted with an overall participation-rate of 30 %, showed no effects on BPSD [14]. As the low level of participation and consequently low level of physical activation could be a determining factor explaining only minimal effects on BPSD, future research should focus on approaches with better exercise adherence and higher levels of physical activation [6].

In addition to physical activation, the organisation of non-pharmacological treatment in the course of a day is a key component of inpatient dementia care [15]. Disease-related changes in circadian motor behaviour, that is, wandering, agitation or sundowning, affect almost every patient in advanced stages of dementia [16]. In this context, sundowning is denoted as an increase of BPSD in the late afternoon and early evening hours. As a low level of daytime activity is linked to a higher fragmentation of circadian rest-activity cycles [17], structured exercise programmes may help to synchronise and reduce circadian rhythm disturbances [16, 18].

Taking into account the assumed positive effects of physical exercise on BPSD and the necessity of providing day structure through activities in dementia care, our research group has conducted a pilot study. The aim of this pilot study was to investigate the feasibility of the planned day-structuring physical activation programme in a hospital setting. Eighteen patients suffering from acute exacerbation of behavioural symptoms in dementia were included. These patients received a 2-week complex day-structuring exercise programme in addition to their usual treatment - the same intervention as planned for the randomised controlled trial (RCT). Two thirds of these patients accomplished more than 180 minutes of exercise per week. In this pilot-study we found reductions in some BPSD; furthermore we found stabilisation of day-structure and a decrease in caregiver burden. Based on these preliminary findings, our planned RCT trial aims to investigate the effects of a day-structuring exercise programme in inpatient hospital dementia care.

### Objectives

The primary objective of this trial is to investigate the effects of a day-structuring exercise programme on BPSD in inpatient hospital dementia care. The promising results of our pilot-study regarding the feasibility and effects of the *exercise-carrousel* on BPSD in hospital old age psychiatry care, led to the following study-hypothesis:

*The intervention group, carrying out a 2-day-structuring exercise intervention in addition to treatment as usual (TAU), is hypothesized to show reduced BPSD at follow-up. In contrast, the control group, receiving social stimulation in addition to TAU, is not expected to show such changes.*

In addition to investigating the putative effects of the day-structuring exercise on BPSD, the following secondary-endpoint parameters are investigated: caregiver burden, total dose and frequency of application of antipsychotic and sedative medication, circadian rhythms of motor behaviour, saliva cortisol-levels and brain-derived neurotrophic factor.

## Methods/Design

### Design

**Fig. 1:**
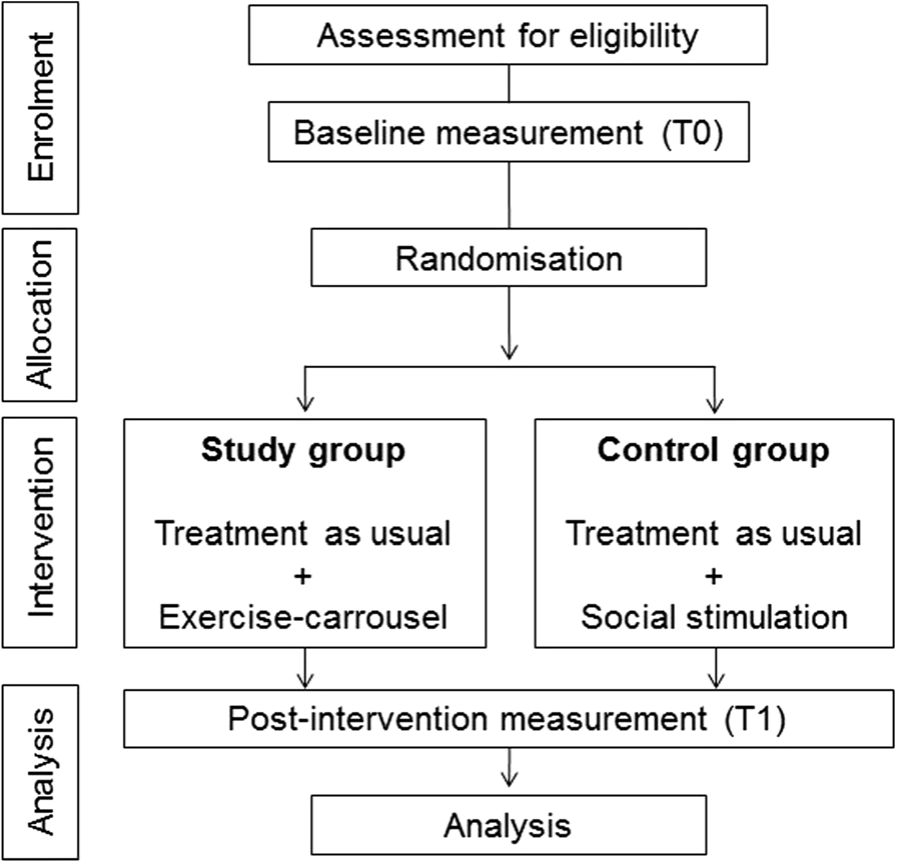
Trial flow chart

An RCT with pre- and post-intervention-assessment (Fig. 1) will be conducted in the department of old age psychiatry at the LVR-Hospital Cologne, an academic teaching hospital of the University of Cologne. All assessors of eligibility criteria and outcome measurements will be blinded to group allocation. Ethical approval was obtained by the Ethics Commission of the German Sport University Cologne and the Ethics Commission of the North-Rhine Medical Chamber (reference number: 2014216). This trial is registered in the German National Register of Clinical Trials (DRKS00006740).

### Sample

Patients from three special DCU of the LVR-Hospital Cologne will be screened by two senior psychiatrists, who are not part of the study team, based on the following inclusion criteria: diagnosis of dementia according to ICD-10-criteria [19] and a minimum length of stay of one week before enrolment into the study, in order to help patients get familiarised to the new ward setting and to exclude delirium. Clinical exclusion of delirium is furthermore based on the validated German version of the Confusion-Assessment-Method, which is performed in all eligible patients [20, 21]. Patients must be able to perform the Timed Up and Go Test (TuG) [22]. Written consent of the patients’ legal guardian as well as from the patient is required. Patients will be excluded according to the following criteria: symptomatic, non-vascular or non-neurodegenerative dementia, no legal guardian, cardiac diseases denying exercise participation; and aggressive behaviour that prevents participation in groups.

### Sample size

The calculation of the sample size was performed via G*Power 3.1 [23], taking into account following parameters: a medium effect size (d = 0.5) for the primary objectives, the overall effects on BPSD measured by the Alzheimer’s Disease Cooperative Study-Clinical Global Impression of Change (ADCS-CGIC) [24, 25], from our pilot-study; a 1:1 allocation rate; two measurements; non-parametric analysis due to ordinal characteristics of the instruments assessing the primary objective; and an α-error of 0.05 and a power of 0.80 (1-ß-error) according to Cohen [26]. Based on this calculation, each group shall include 53 patients. The enrolment of 130 patients is planned, as a drop-out rate of 25 % is expected based on the experience from the pilot study.

#### Allocation and concealment of allocation

After enrolment and pre-assessment (T0, Fig. 1), group allocation to the study or control group will be realised via minimisation. An external statistician, who is not part of the study team, will run the group allocation via an online minimisation-tool ‘Qminim’ [27]. Minimisation is a well-approved form of randomisation that minimises group differences in pre-defined characteristics while continuing trial enrolment. In order to achieve the best possible balance between the two study arms, group allocation will be performed according to the following factors: sex (double weighted), age, Mini-Mental Status-Examination (MMSE) [28] and Bayer instrumental Activities of Daily Living (B-ADL) [29]. Due to external enrolment in the trial, central randomisation by a person not involved in the study, and the blinding of all investigators during the whole trial, allocation concealment will be realised in the course of the trial.

#### Sample characteristics

Baseline characteristics will be recorded within the geriatric assessment, carried out by nursing and medical staff members of the LVR-Hospital Cologne, who are not part of the study team. This basic assessment includes sex, age, body-mass-index, type of dementia, the MMSE [28], the clock draw test [30], assessment of the cognitive reserve capacity according to Stern [31], TuG [22], the 10-meter gait speed [32] and B-ADL [29].

#### Outcome measures

All outcome measurements will be conducted by experienced assessors from nursing and medical staff, blinded to group allocation. Primary and secondary outcome variables are presented in Table 1.

**Table 1:** Objectives and instrumental set-up of the trial

	Variable	Instrument
Primary objective	Behavioural and psychological symptoms (BPSD)	Alzheimer’s Disease Cooperative Study-Clinical Global Impression of Change [33]
Secondary objective	Dimensions of BPSD	Neuropsychiatric Inventory (NPI) [35]
		Cohen-Mansfield-Agitation-Inventory [36]
	Caregiver burden	NPI-caregiver-subscale [35]
	Application of antipsychotics	Chlorpromazine-equivalents [34]
	Application of benzodiazepine	Diazepam-equivalents [36]
	Motor behaviour	Body fixed motion sensor
	Saliva cortisol levels	Demeditec™ saliva kits [42]
	Neurotrophic factor levels	Brain-derived neurotrophic factor from blood-serum probes [44]

As the overall effect of our intervention on BPSD as rated by the ADCS-CGIC [24, 25] is the primary outcome, that is the measure on which we will focus.

**Fig. 2:**
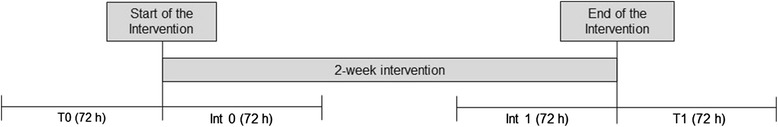
Schedule of body-fixed sensor assessment of patient motor behaviour. Legend: Four 72-h episodes at baseline (T0); at the beginning of the intervention (Int 0); at the end of the intervention (Int 1) and after the intervention (T1)

Secondary outcome measures include dimensions of BPSD rated by the Neuropsychiatric Inventory (NPI) [33] and the Cohen-Mansfield-Agitation-Inventory (CMAI) [34]. All psychopathometric assessments will be accomplished by blinded investigators, who are responsible for interviewing both the caregiver as well as the medical and nursing staff. Further secondary outcome variables include the assessment of caregiver burden via the NPI-caregiver-subscale [33] and the effects on the application of two categories of psychotropic medication: antipsychotics and benzodiazepines. The individual dosage per day of routine and pro-re-nata medication will be converted for antipsychotics to Chlorpromazine equivalents [35] and for benzodiazepines to Diazepam-equivalents [36, 37]. The patients’ motor behaviour will be assessed by an inertial body-fixed sensor system. Body postures will be detected in four periods of 72 consecutive hours (see Fig. 2), based upon accelerometer, gyroscope, and magnetoscope data. As recommended by Trost *et al.* [38], the sensor system will be placed at the nearest possible position to the body centre of mass, the lower back. In accordance to Hatfield *et al.* [39], diurnal saliva cortisol-levels will be assessed every four hours from 08:00 a.m. to 12:00 p.m. on two consecutive days at T0 and T1 with Demeditec™ saliva kits (Demeditec Diagnostics GmbH, Kiel, Germany) [40]. Analysis of saliva cortisol-levels will be carried out via an enzyme-linked immunosorbent assay (ELISA). In order to analyse the effects on brain-derived neurotrophic factor (BDNF), blood-samples will be taken on the first intervention day at 08:30 a.m., at 05:00 p.m. and for T1 at 08:30 a.m. These samples will be centrifuged, blood serum will be stored in a fridge by -40 °C and the ELISA analysis will be realised via R & D Systems™ (R&D Systems Inc., Minneapolis, USA) [41–43]. Adverse events are recorded and a potential relation to the intervention will be evaluated by a senior old age psychiatrist, who is not part of the study team.

#### Intervention

TAU includes exercise therapy for 45 minutes twice weekly. Patients within the study group will receive a 2-week exercise programme. On 3 non-consecutive days of the week, the patients allocated to the study-group will be offered four exercise sessions a day (see Table 2). In order to ease the patients’ participation, the exercise instructor will see the patient on his ward and ask him to take part in an exercise session. If the patient refuses or is hindered for other reasons, the exercise instructor will, however, see the patient at the next scheduled exercise session (Table 2) and offer him further participation. After positive feedback, a single session will be held for a net time of 20 minutes. After one exercise session, there will be a rest period of one hour. This specific time schedule will be carried out four times on each intervention day. Due to the provision of recurrent rest-activity periods throughout a day, this activation programme is called *exercise-carrousel.* The patients will be allocated to three different exercise groups with a maximum of three patients per instructor. Two exercise sessions include endurance exercise on seated-ergometers for the upper and lower limb. Furthermore, two exercise sessions focusing on strengthening exercises with wrist- and ankle-worn weights will be carried out. The net exercise time of 20 minutes includes two minutes of warm-up at the beginning and two minutes of cool down at the end. An endurance-exercise-period of 16 minutes or alternatively six strengthening exercises will be carried out.

**Table 2:** Exercise carrousel schedule for one intervention day

Time/Group	a.m.						p.m.						
	09:00	09:30	10:00	10:30	11:00	11:30	00:00 to 02:00	02:00	02:30	03:.00	03:30	04:00	04:30
Group 1	I			II			Lunch break	III			IV		
Group 2		I			II				III			IV	
Group 3			I			II				III			IV

Strengthening sessions include six standardised strengthening exercises for the lower limb with two to five kilogramme ankle-worn weights on both legs (sit-stand, knee-extension in sitting position, standing heel-raise, knee-curl in standing position, hip-abduction and hip-extension in standing position); for the upper limb with 1- to 2-kg wrist-worn weights on both arms (biceps-curl, extended shoulder-lift in sitting position, rowing in 90° shoulder-abduction, shoulder-lift and shoulder-abduction in standing, dips on arm rest while sit-stand).

These strengthening exercises will run in two sets of twelve repetitions each. The exercise load will be increased progressively after each intervention day in increments of 1 kg for the lower limb and a 0.5 kg for the upper limb. An increase in exercise load requires optimal exercise execution regarding both range of motion and scheduled repetitions. Endurance exercise sessions are conducted with a scheduled intensity of one watt per kilogramme of body weight in upper and lower limb exercises. If the exercise sessions a) are adequately executed and b) no serious complications occurred, the exercise load will be augmented up to 1.5 watt per kilogramme body weight. In accordance to Edwards *et al.* [44] exercise adherence will be recorded for each exercise session, and not only physical presence will be considered but also the degree to which the scheduled exercise session was completed. For participation to be rated as complete, more than 50 % of the scheduled exercise load regarding time, intensity and adequate repetitions is required.

The average participation rate of subjects in our pilot study was 120 min/week. For our RCT, we expect the same average participation duration for the intervention group. In order to receive a comparable level of social stimulation to the study group, the patients in our control group will be offered two 1-hour sessions of social interaction (table games) per week.

#### Analysis

The patients’ baseline characteristics in both groups will be compared via Chi-square test for categorical data, Wilcoxon rank test for non-parametric or non-normally distributed characteristics and *T*-test for continuous and normally distributed characteristics. Pre-post-outcome analysis within and between the groups will be realised per intention-to-treat analysis for the primary outcome variable via Friedman analysis of variance (ordinal characteristics) and analysis of variance for interval characteristics. The analysis of circadian rhythms in motor behaviour will be conducted according to Witting *et al.* [45] by calculating inter-daily stability, intra-daily variability and relative amplitude. Advanced time series analysis of the patients’ daily activity patterns will be developed in accordance with recent developments by Kirste *et al.* [46]. Analysis of the patients’ characteristics and outcome criteria will be realised using SPSS (version 22) and Matlab (version R2014b).

## Discussion

The treatment of BPSD is challenging as patients with dementia are prone to suffer from severe side effects of pharmacotherapy [47]. Furthermore, psychopharmacotherapy often has no significant effects on BPSD or may even worsen behavioural symptoms in the patients [48]. Therefore, non-pharmacological approaches for the treatment of BPSD are essential. Establishing non-pharmacological treatment and especially physical activation therefore represent a fruitful and promising approach [5]. Hence, we plan to implement a hospital-based day-structuring exercise programme for demented patients suffering from exacerbated behavioural symptoms.

To our knowledge, this is the first randomised controlled trial in inpatient hospital dementia care investigating the effects of multiple exercise sessions in the course of a day. Whereas former intervention trials and routine inpatient dementia care provide exercise sessions in a rigid time schedule for approximately one hour per session [14], this trial offers four flexible short-time exercise sessions throughout a day. In a hospital setting, ward routines and intra-daily variations of the patients’ mood or behaviour are most reasons for non-participation in exercise sessions. If a patient has no motivation to take part in an exercise session, or is hindered due to nursing or medical routines, there is in most cases no further chance to take part in guided physical activation throughout the same day. In order to circumvent these problems, the *exercise-carrousel* schedule offers multiple low-threshold exercise sessions during a day. From our clinical experience, this exercise programme should increase the patients’ willingness for participation. Therefore, we expect a better exercise-adherence compared to routine care and former trials focussing on physical activation in dementia [14]. In accordance with Christofoletti *et al.* [6], this programme represents an innovative strategy to increase exercise-adherence in dementia patients, thereby possibly leading to decreased BPSD.

Additionally, we are also interested in the influences on circadian rhythm disturbances. Disrupted circadian motor behaviour is often seen in demented patients; that is, fragmentation of rest-activity cycles or wandering-phenomenon. Actually, there are recommendations to perform exercise sessions in the morning hours [49]. As behavioural disturbances more often occur in the afternoon and evening hours, Rimmer and Smith [49] were more concerned with behavioural disturbances that may intervene with exercise sessions at that time. In most hospital and nursing home settings, physical and occupational therapy is therefore centred in the morning hours. Consequently, in daily routines for dementia patients there is often a lack of guided activities in the afternoon and evening hours. National guidelines and international expert panels demand the provision of day structure as non-pharmacological treatment of circadian rhythm disruptions [15, 50, 51]. To our knowledge, no trial focussing on the provision of day structure through physical activation in dementia care has been conducted so far [15, 51].

We plan a trial that provides structured rest- and activity-sequences in the course of a day and investigates the synchronising effects on circadian rhythm disturbances. Intervention days are planned on three non-consecutive days a week in addition to usual care with daily co-therapies, nursing and medical applications. To our knowledge there is no evidence, and neither are there recommendations, for daily structured activities as treatment of circadian rhythm disturbances. Within the thrice weekly exercise schedule, we expect a day of rest to be necessary for the patients and for the evaluation of exercise effects on circadian disruptions on intervention and non-intervention-days. Based on the experiences from our pilot project, a day of rest between the intervention days seems to be useful for these older population patients.

In order to appropriately assess behavioural symptoms, circadian rhythm disruptions and the effects of the day-structuring exercise schedule, we suggest an innovative combination of body-fixed motion sensors, well-established instruments for the rating of BPSD (NPI, CMAI) and physiological parameters (saliva-cortisol, BDNF). The upcoming complementary use of hybrid motion sensors is an especially important methodological advancement evaluating the association between disrupted motor activity and behavioural symptoms in dementia [46]. Wrist-worn accelerometers, logging and analysing the periodicity of limb movements on the basis of activity counts, are usually used to investigate movement behaviour and circadian rhythm disruptions [52]. Sensors logging the periodicity of body postures, may be superior to activity counts in analysing these symptoms. The results of our planned trial may help to implement and improve the clinical use of body-fixed sensors in assessing circadian and behavioural symptoms in patients suffering from dementia [46, 53].

Considering the methodological issues, a major challenge in conducting clinical trials is to prevent selection bias and achieve allocation concealment for all investigators throughout the whole trial. Therefore, all investigators involved in the assessment of patients (BPSD, motor behaviour, cortisol, BDNF) and the staff of the ward are blinded to group allocation. Trial design as well as the definition and recording of intervention adherence are important features when conducting exercise trials in patients with dementia. In accordance to Edwards *et al.* [44], we will rate exercise adherence regarding time, intensity and degree of exercise performance. We do not plan to use standardised self-reported rating scales to assess exercise exertion (for example, via Borg score [54]), as these scores are not reliable in patients with advanced dementia [55]. Apart from the application of body-fixed motion sensors, which provide reliable and objective information about motor behaviour during participation in exercise sessions, there is no further monitoring of exercise exertion planned for this trial. On the one hand, the monitoring of heart rate and blood pressure during exercise participation has not been feasible during the pilot project. These patients were often distracted and sometimes refused to wear the monitoring devices. On the other hand, we experienced small motor sensors fixed on the lower back to be out of the patients’ focus and therefore to be well accepted. The lack of instrumental monitoring of exercise exertion represents a limitation of this trial. Because this pragmatic method favours the provision of best possible low-threshold exercise participation, with the lowest possible obstruction before and during an exercise session, this approach has possible methodological limitations, but it is also a simple, transferable approach for routine care. Furthermore, the 2-week intervention phase only allows investigation into the short-term effects of the exercise programme. Taking into account the regular stay length of 4 to 5 weeks in inpatient old age psychiatric hospital care, the design of this trial schedules a 2-week intervention phase, due to 1 week after admission for familiarisation with the setting and delirium exclusion, and 3 days for pre- and post-assessment, respectively. Apart from the usually recommended long-term exercise schedules, the investigation of an intensive short-term *exercise-carrousel* programme represents, from our point of view, an appropriate and easily transferable approach at this stage of care. Obviously, this intensive exercise intervention programme requires a greater use of therapy resources and may negatively interact with other hospital routines.

Physical activation is a key part of non-pharmacological treatment, but despite the clinicians’ advice to apply non-pharmacological methods for the treatment of BPSD [4], there are no recommendations and guidelines for inpatient hospital dementia care. The results of our study may help to increase the evidence in the field of exercise science and non-pharmacological treatment. The *exercise-carrousel* approach can further advance non-pharmacological treatment, leading to potential worthwhile developments for patients, medical staff and caregivers in dementia care.

### Trial status

Recruitment began in April 2015

## Abbreviations

ADCS-CGIC: Alzheimer’s Disease Cooperative Study-Clinical Global Impression of Change
B-ADL: Bayer instrumental activities of daily living
BDNF: brain-derived neurotrophic factor
BPSD: behavioural and psychological symptoms of dementia
CMAI: Cohen-Mansfield agitation inventory
DCU: dementia care unit
ELISA: enzyme-linked immunosorbent assay
MMSE: mini-mental status-examination
NPI: neuropsychiatric inventory
RCT: randomised controlled trial
TAU: treatment as usual
TUG: timed up and go-test
